# Infective endocarditis leading to pulmonary pseudoaneurysm and severe pulmonary valve regurgitation: A case report

**DOI:** 10.1097/MD.0000000000043910

**Published:** 2025-08-08

**Authors:** Qin Yin, Qi An

**Affiliations:** a Department of Anesthesiology, West China Hospital, Sichuan University/West China School of Nursing, Chengdu, Sichuan, China; b Department of Cardiovascular Surgery, West China Hospital, Sichuan University, Chengdu, Sichuan, China.

**Keywords:** conduit placement, congenital heart disease, infective endocarditis, pseudoaneurysm

## Abstract

**Rationale:**

Pulmonary pseudoaneurysm resulting from infective endocarditis is a rare complication in patients with congenital heart disease who have undergone conduit placement.

**Patient concerns:**

In this article, we report a rare case of late-onset bacterial infective endocarditis complicated by a pseudoaneurysm and severe pulmonary valve regurgitation in a 16-year-old girl with double-outlet right ventricle, ventricular septal defect, and pulmonary stenosis, who had previously undergone a Rastelli procedure at age 15.

**Diagnoses:**

Based on the patient’s medical history and transthoracic echocardiography examinations and cardiac computed tomography angiography, a definitive diagnosis of late-onset bacterial infective endocarditis complicated by a pseudoaneurysm and severe pulmonary valve regurgitation was established.

**Interventions:**

Given the patient’s worsening clinical condition, surgery for ventricular septal defect closure, placement of a new right-ventricle-to-pulmonary-artery conduit with a Gore-tex conduit was conducted.

**Outcomes:**

The surgical procedure was successful. The patient was extubated 3 hours postoperatively and received 6 weeks of antibiotic therapy postoperatively, during which no further episodes of fever occurred, and the inflammatory markers decreased to normal levels.

**Lessons:**

For bacterial infective endocarditis associated with pulmonary pseudoaneurysm and severe pulmonary valve regurgitation, early surgical intervention is essential to mitigate further cardiac dysfunction and improve clinical outcomes.

## 1. Introduction

Infective endocarditis (IE) is a relatively uncommon condition in the pediatric population. As a result, there is a notable scarcity of literature that specifically addresses cases involving unique or resistant organisms. The complications associated with infective endocarditis are not only rare but also poorly characterized in medical literature. This lack of detailed information is particularly evident when it comes to unusual sequelae, such as the formation of pseudoaneurysms and the development of severe pulmonary valve regurgitation. These conditions, while rare, pose significant challenges in diagnosis and treatment, highlighting the need for further research and documentation in this area.^[1]^ IE is a severe and potentially fatal condition, with in-hospital mortality rates between 15% and 30%. Perivalvular extension is the most common cause of persistent infection following IE, occurring in up to one-third of patients who undergo systematic transesophageal echocardiography screening. Effective treatment of this condition depends on the eradication of the causative microorganisms using antimicrobial drugs, often in combination with surgical intervention to remove the infected tissue.^[2]^ Pulmonary artery pseudoaneurysms are rare but carry a high mortality risk. If left untreated, these lesions may enlarge and rupture, causing fatal bleeding. Clinical presentations vary widely, from life-threatening hemorrhages to asymptomatic lesions that can grow over extended periods, spanning days, months, or even years. Early detection and treatment are crucial, often facilitated by imaging studies. Embolization is typically the preferred treatment method. Radiologists play a vital role in both the timely diagnosis and management of pulmonary artery pseudoaneurysms. Pseudoaneurysms resulting from bacterial endocarditis or complications related to pulmonary artery catheters and right heart catheterization may require surgical intervention.^[3]^ We reported a rare case of late-onset bacterial infective endocarditis complicated by a pseudoaneurysm and severe pulmonary valve regurgitation in a 16-year-old girl with double-outlet right ventricle, ventricular septal defect (VSD), and pulmonary stenosis, who had previously undergone a Rastelli procedure at age 15.

## 2. Case report

A 16-year-old female, with a known diagnosis of double-outlet right ventricle, VSD, and pulmonary stenosis, underwent a Rastelli procedure at 15 years of age, during which a 20 mm handmade trileaflet-valved extracardiac Gore-Tex conduit was implanted. The procedure was initially successful, and the patient experienced an uneventful postoperative recovery. However, 1 year later, she began to experience recurrent episodes of fever, which persisted despite antibiotic treatment.

**Figure 1.**
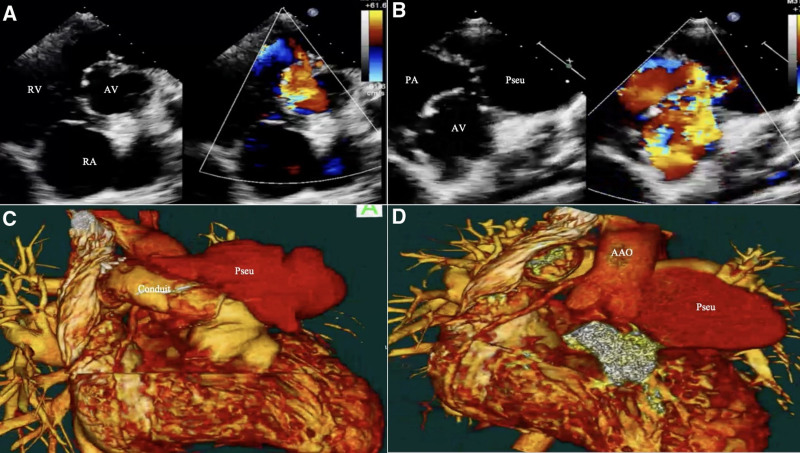
A and B showed severe pulmonary valve regurgitation, a residual VSD patch leakage (4 mm), moderate tricuspid regurgitation, suspicion of infective endocarditis (IE). C and D confirmed the diagnosis of pulmonary pseudoaneurysm. VSD = ventricular septal defect.

Upon admission to our hospital, the patient presented with persistent fever, respiratory distress, facial puffiness and swelling of bilateral legs and elevated inflammatory markers. Transthoracic echocardiography revealed severe pulmonary valve regurgitation, a residual VSD patch leakage (4 mm), moderate tricuspid regurgitation, and suspicion of IE (Fig. 1A and B). Additionally, a pulmonary pseudoaneurysm was noted. Cardiac computed tomography angiography was performed, which confirmed the presence of a pulmonary pseudoaneurysm (Fig. 1C and D). This was believed to be the result of ongoing infective endocarditis. Given the patient’s worsening clinical condition, surgery for VSD closure, placement of a new right-ventricle-to-pulmonary-artery conduit with a Gore-tex conduit was scheduled.

**Figure 2.**
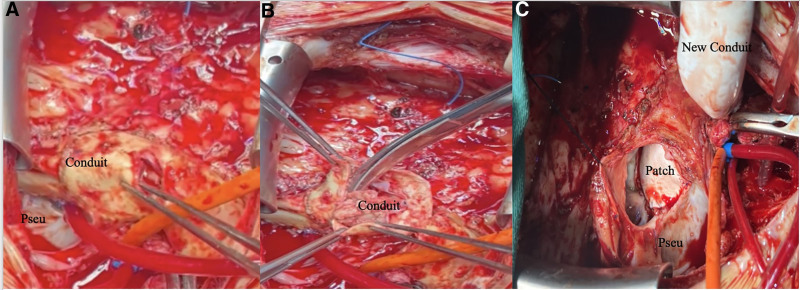
A and B showed pulmonary pseudoaneurysm located on the left side of the pulmonary artery root and a residual VSD patch leakage beneath the pulmonary valve. C showed a new Gore-Tex patch used to close the VSD, and a new 22-mm polytetrafluoroethylene conduit was implanted to connect right ventricle and pulmonary artery. VSD = ventricular septal defect.

The patient was initially catheterized via the femoral artery and vein to establish cardiopulmonary bypass. After securing the aortic cross-clamp, the (right-ventricle-to-pulmonary-artery conduit) was incised, Infective endocarditis was identified within the conduit which was severely damaged by multiple bacterial vegetation. The pulmonary pseudoaneurysm which located on the left side of the pulmonary artery root was found to be approximately 30 mm in diameter. Additionally, a residual VSD patch leakage was noted beneath the pulmonary valve (Fig. 2A and B). The VSD patch was removed, and the defect was subsequently closed using a new Gore-Tex patch, and a new 22-mm polytetrafluoroethylene conduit was implanted (Fig. 2C). Postoperative hemodynamic measurements indicated a right ventricular pressure of 35/2 mm Hg and a systemic blood pressure of 102/55 mm Hg. The surgical procedure was successful. The patient was extubated 3 hours postoperatively and received 6 weeks of antibiotic therapy postoperatively, during which no further episodes of fever occurred, and the inflammatory markers decreased to normal levels. A follow-up echocardiogram performed 3 months after discharge demonstrated mild pulmonary valve regurgitation without vegetation and no residual VSD shunt.

## 3. Discussion

IE is a bacterial or fungal infection that affects the endocardium or heart valves. Patients with congenital heart disease (CHD) are at a significantly higher risk of developing IE, with their risk being 15 to 50 times greater than that of the general population. This elevated risk is mainly attributed to the presence of prosthetic materials and residual lesions, and such patients account for up to 18% of all IE cases.^[4,5]^ Approximately 50% of children with IE complicating CHD have undergone prior cardiac surgery, particularly palliative shunt procedures or complex intracardiac repairs. Research indicates that the highest risk for IE is among children with cyanotic CHD, especially those who have had surgery for pulmonary blood flow obstruction or prosthetic aortic valve replacement.^[6]^

Even with appropriate medical treatment, IE can lead to severe complications, such as valvular dysfunction, abscess or fistula formation. Pulmonary pseudoaneurysm, a rare but significant complication of IE, is particularly notable in patients with CHD who have undergone conduit placement. This condition arises from a disruption in the vascular wall, usually caused by bacterial invasion that weakens the arterial structure. In patients who have had the Rastelli procedure, the implanted conduit can become a site of infection, potentially leading to pulmonary pseudoaneurysm. In this case, the patient developed severe pulmonary valve regurgitation, a pulmonary pseudoaneurysm, and a residual VSD shunt, complicating her clinical presentation.^[7]^

For patients with such complex conditions, the treatment approach generally encompasses a multifaceted strategy that combines antibiotic therapy to effectively manage and control the underlying infection, along with surgical intervention to address the pulmonary pseudoaneurysm and any associated conduit dysfunction. In cases where patients exhibit persistent sepsis, large mediastinal abscesses, or mycotic pseudoaneurysms, early surgical intervention is often recommended to mitigate the severity of the condition and prevent further complications.^[8]^

Once the infective endocarditis has been successfully resolved, the removal of the cardiac lesion may be considered. This step is crucial as it helps to eliminate the source of infection and significantly reduces the potential risk of recurrence. It is important to note that continued antimicrobial therapy remains an essential component of the postoperative management plan for these patients. This ongoing treatment is vital for several reasons: it helps to ensure that any residual infection is completely eradicated, it prevents the recurrence or reactivation of infective endocarditis, and it ultimately contributes to reducing the risk of postoperative complications. By maintaining a robust antimicrobial regimen, healthcare providers can enhance the long-term outcomes for these patients, promoting better overall health and quality of life.

## 4. Conclusion

We report a rare case of bacterial IE associated with pulmonary pseudoaneurysm and severe pulmonary valve regurgitation in a 16-year-old girl. This case emphasizes the critical importance of vigilant, long-term follow-up in patients with complex CHD and the need for prompt intervention in the face of persistent systemic infection and structural abnormalities. Early surgical intervention is essential to mitigate further cardiac dysfunction and improve clinical outcomes.

## Author contributions

**Conceptualization:** Qin Yin, Qi An.

**Data curation:** Qin Yin, Qi An.

**Formal analysis:** Qin Yin, Qi An.

**Investigation:** Qin Yin, Qi An.

**Methodology:** Qin Yin, Qi An.

**Project administration:** Qin Yin, Qi An.

**Resources:** Qin Yin, Qi An.

**Software:** Qin Yin, Qi An.

**Supervision:** Qin Yin, Qi An.

**Validation:** Qin Yin, Qi An.

**Visualization:** Qin Yin, Qi An.

**Writing – original draft:** Qin Yin, Qi An.

**Writing – review & editing:** Qin Yin, Qi An.
